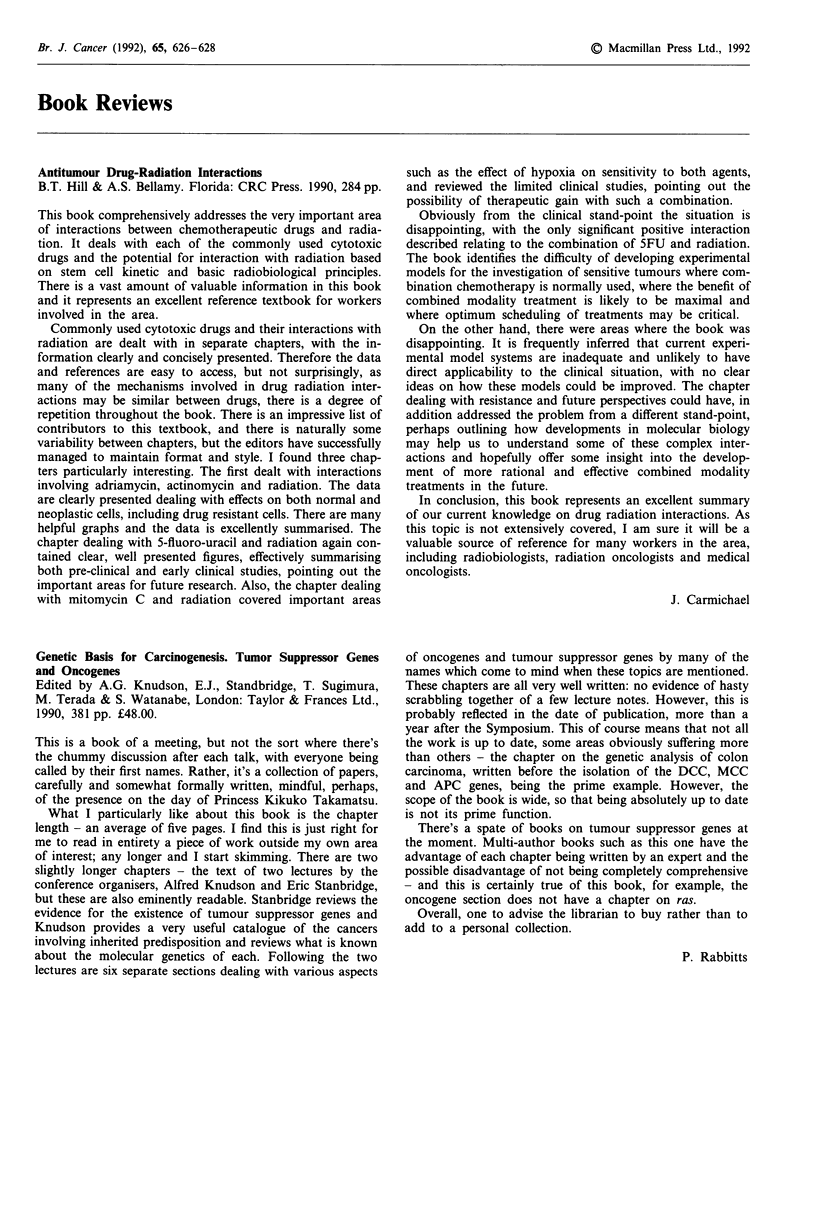# Genetic Basis for Carcinogenesis. Tumor Suppressor Genes and Oncogenes

**Published:** 1992-04

**Authors:** P. Rabbitts


**Genetic Basis for Carcinogenesis. Tumor Suppressor Genes and Oncogenes**
Edited by A.G. Knudson, E.J., Standbridge, T. Sugimura, M. Terada & S. Watanabe, London: Taylor & Frances Ltd., 1990, 381 pp. £48.00.

This is a book of a meeting, but not the sort where there's the chummy discussion after each talk, with everyone being called by their first names. Rather, it's a collection of papers, carefully and somewhat formally written, mindful, perhaps, of the presence on the day of Princess Kikuko Takamatsu.

What I particularly like about this book is the chapter length – an average of five pages. I find this is just right for me to read in entirety a piece of work outside my own area of interest; any longer and I start skimming. There are two slightly longer chapters – the text of two lectures by the conference organisers, Alfred Knudson and Eric Stanbridge, but these are also eminently readable. Stanbridge reviews the evidence for the existence of tumour suppressor genes and Knudson provides a very useful catalogue of the cancers involving inherited predisposition and reviews what is known about the molecular genetics of each. Following the two lectures are six separate sections dealing with various aspects of oncogenes and tumour suppressor genes by many of the names which come to mind when these topics are mentioned. These chapters are all very well written: no evidence of hasty scrabbling together of a few lecture notes. However, this is probably reflected in the date of publication, more than a year after the Symposium. This of course means that not all the work is up to date, some areas obviously suffering more than others – the chapter on the genetic analysis of colon carcinoma, written before the isolation of the DCC, MCC and APC genes, being the prime example. However, the scope of the book is wide, so that being absolutely up to date is not its prime function.

There's a spate of books on tumour suppressor genes at the moment. Multi-author books such as this one have the advantage of each chapter being written by an expert and the possible disadvantage of not being completely comprehensive – and this is certainly true of this book, for example, the oncogene section does not have a chapter on *ras*.

Overall, one to advise the librarian to buy rather than to add to a personal collection.

P. Rabbitts